# Weight-Based Health Care Discrimination and Cervical Cancer Screening Among Black Sexual and Gender Minoritized Assigned Female at Birth Adults in the United States

**DOI:** 10.1089/heq.2024.0158

**Published:** 2025-01-13

**Authors:** Simran Singh, Neil Mehta, Madeline Noh, Keosha Bond, Megan Threats, John W. Jackson, Nkiru Nnawulezi, Marquisele Mercedes, Madina Agénor

**Affiliations:** ^1^ Department of Behavioral and Social Sciences, Brown University School of Public Health, Providence, Rhode Island, USA.; ^2^ Center for Health Promotion and Health Equity, Brown University School of Public Health, Providence, Rhode Island, USA.; ^3^ Department of Community Health and Social Medicine, CUNY School of Medicine at The City College of New York School of Medicine, New York, New York, USA.; ^4^ School of Information, University of Michigan, Ann Arbor, Michigan, USA.; ^5^ Department of Epidemiology, Johns Hopkins Bloomberg School of Public Health, Baltimore, Maryland, USA.; ^6^ Department of Psychology, University of Maryland, Baltimore County, Baltimore, Maryland, USA.; ^7^ Department of Behavioral and Social Sciences, Brown University, Providence, Rhode Island, USA.; ^8^ Department of Epidemiology, Brown University School of Public Health, Providence, Rhode Island, USA.

**Keywords:** weight discrimination, LGBT, cervical cancer screening, gender identity, sexual identity

## Abstract

**Introduction:**

Black sexual and gender minoritized (SGM) people assigned female at birth (AFAB) face notable barriers to cervical cancer screening, including racism, heterosexism, and cisgenderism. Although weight-based discrimination is prevalent in the United States and may compound other forms of discrimination, no study has examined the association between weight-based discrimination in health care settings and Pap test use among Black SGM AFAB.

**Materials and Methods:**

We conducted a cross-sectional online survey among Black SGM AFAB adults aged 18–45 years (*N* = 135) and used multivariable logistic modeling to analyze the association between weight-based health care discrimination and Pap test use, adjusting for demographic, socioeconomic, and health care factors.

**Results:**

Approximately one quarter (27.5%; *n* = 33) of respondents eligible for a Pap test had ever experienced weight-based health care discrimination. Moreover, 63.3% (*n* = 76) and 45% (*n* = 54) of respondents had ever received a Pap test in their lifetime and in the last 3 years, respectively. Respondents who had experienced weight-based health care discrimination had significantly lower adjusted odds of having ever received a Pap test in their lifetime (odds ratio [OR] = 0.10; 95% confidence interval [CI]: 0.02–0.40) and in the last 3 years (OR = 0.07; CI: 0.01–0.31) compared with those who had never experienced such discrimination.

**Discussion:**

Additional research is needed to elucidate the unique experiences of specific subgroups of Black SGM people and to inform policies, norms, and practices that mitigate the occurrence and effects of weight-based health care discrimination among Black SGM people in the context of cervical cancer screening and other health services.

## Introduction

Research examining the sexual and reproductive health experiences of Black sexual and gender minoritized (SGM; e.g., lesbian, bisexual, queer, transgender, non-binary) people who were assigned female at birth (AFAB) is scarce. However, there is a long history of Black AFAB people,^1–4^ as well as SGM individuals,^5,6^ experiencing mistreatment, coercion, and exploitation in the context of sexual and reproductive health care (SRH). Existing research shows that, in the United States, Black SGM AFAB people are disproportionately impacted by adverse sexual and reproductive health outcomes^7–10^ and face notable barriers to high-quality SRH, including a lack of access to health insurance and a usual source of care,^9,11^ socioeconomic inequities,^9,12^ health care provider discrimination,^7,9,10,13–16^ and discordance between patient and provider sociodemographic background, undermining patient-provider communication.^10^

Routine cervical cancer screening is an essential aspect of SRH that allows for the early detection and secondary prevention of cervical cancer.^17^ Studies indicate that cervical cancer incidence rates may be higher among lesbian and bisexual women compared with heterosexual women.^18,19^ Moreover, research shows that Black women are also more likely than white women to be diagnosed with and die from cervical cancer.^20,21^ Rates of cervical cancer morbidity and mortality are unknown for Black SGM AFAB individuals in particular. However, researchers have found that Black SGM AFAB individuals experience pronounced barriers to cervical cancer screening which may increase their risk of cervical cancer as a result of racism, sexism, classism, and heterosexism,^9–11,13,14^ including biased health care provider assumptions about Black SGM AFAB people’s sexual behavior and capacity to understand health and poor patient-provider communication.^10^ Similarly, other studies indicate that Black transgender men and non-binary AFAB people experience racism, cissexism, and heterosexism when accessing Pap testing,^8,22^ including hyper-sexualization, racist provider beliefs about pain tolerance, and provider refusal to affirm their gender identity.^22^ Further, transgender men and non-binary AFAB people are not mentioned or included in cervical cancer screening guidelines, although they follow the same recommendations as cisgender women;^23,24^ this exclusion may pose an additional barrier to receiving routine Pap testing.

Weight-based discrimination—that is, discrimination toward individuals due to body weight or size—is a highly prevalent yet understudied aspect of discrimination that affects a large number of U.S. adults,^25–27^ especially Black^16,28–30^ and SGM^16,28,31,32^ individuals. Interdisciplinary scholarship demonstrates that weight-based discrimination—rooted in anti-fatness, anti-Blackness, and heteropatriarchy—is co-constituted with other forms of discrimination, including racism, sexism, heterosexism, and cisgenderism.^33–35^ Further, clinician use of body mass index (BMI) may perpetuate weight-based discrimination by upholding whiteness as the medical standard and not accounting for differences across racial/ethnic groups.^34,36^ To counteract biases, fat studies have reclaimed the term “fat” as a neutral descriptor, replacing stigmatizing terms like “obese” or “overweight” based on BMI.^37^ This stigma and weight-based discrimination in health care may undermine fat people’s bodily autonomy and dignity,^33,34^ having been linked to a range of adverse sexual and reproductive health outcomes, including a lack of routine gynecological care, unmet contraceptive needs, refusal of fertility treatment, negative maternity care experiences, and poor birthing outcomes (e.g., pre-term birth, miscarriage).^16,30,32,38–41^

However, few studies on weight-based discrimination in the context of SRH in general or cervical cancer screening, in particular, have addressed the unique and specific experiences of Black SGM AFAB people,^22,28,32^ a multiply marginalized population among whom the effects of discrimination based on weight may be compounded by racism, sexism, heterosexism, and cisgenderism.^33–35^ Thus, we designed an exploratory quantitative study to examine the association between weight-based discrimination and Pap test use among Black SGM AFAB people in the United States, with the goal of informing future research, policies, and interventions that help combat weight-based discrimination in health care settings and facilitate access to high-quality preventive health care among Black SGM AFAB people.

## Materials and Methods

We purposively sampled Black sexual (e.g., lesbian) and gender (e.g., transgender) minoritized (SGM) U.S. adults aged 18–45 years who were AFAB to complete a cross-sectional online survey in January and February 2023. The survey was posted on the online survey platform Prolific, which has assembled an existing panel of pre-screened potential participants; only those who met study eligibility criteria were shown the survey.^42^ This process yielded a final sample of 156 respondents. Participants who were deemed eligible in the screening survey were provided with a unique Qualtrics link to complete the main survey. To be eligible, participants needed to be AFAB, identify with an SGM identity, aged 18–45 years, identify as Black, African American, African, or Caribbean, and live in the United States or a U.S. territory. Participants provided written informed consent at the start of the survey and received $20 per hour for their participation, in addition to sexual and reproductive health information and resources upon completion of the survey. All research activities were approved by the Brown University Institutional Review Board.

The primary predictor was ever experiencing weight-based discrimination in health care settings (yes/no), which was assessed using the Discrimination in Medical Settings (DMS) Scale.^43^ Participants who responded “yes” to ever experiencing any discrimination in a health care setting and selected the answer “weight” to the question “What do you think is the main reason for these experiences?” were categorized as having experienced weight-based health care discrimination. The binary outcomes of interest were having received a Pap test in the last 3 years and ever having received a Pap test. The Pap test in the last 3 years variable was modeled after the 2017–2019 National Survey of Family Growth measure for comparison purposes.^44^ Guidelines by the U.S. Preventive Services Task Force recommend individuals aged 21–29 years complete cervical cancer screening at least every 3 years and individuals aged 30–65 every 3–5 years based on the test^45^; therefore, participants under the age of 21 were excluded from our analytic sample. Other variables were included in the model, listed in Table 1, that were organized in theoretically informed sets (i.e., demographic, socioeconomic, and health care factors), with the order based on our conceptualization of demographic,^9,10,15,46,47^ socioeconomic (SE),^46,48–50^ and health care (HC)^51–53^ factors as potential covariates related to both weight-based discrimination and Pap test use. Specifically, we entered factors in a stepwise manner (i.e., demographic factors first, followed by SE factors, then HC factors) to qualitatively ascertain how each set influenced the association between our predictor and outcome variables. Demographic factors were entered before SE factors to first account for fixed population characteristics. SE factors were then entered before HC factors to determine whether HC factors, adjusting for the less modifiable socioeconomic factors, mitigated the observed association.

**Table 1.** Distribution of Demographic, Socioeconomic, and Health Care Factors Among Black Sexual Minoritized Cisgender Women, Transgender Men, and Non-Binary Assigned Female at Birth U.S. adults (*N* = 120)

Variable	*n*	%
Age		
21–29	76	63.3
30–45	44	36.7
Sexual orientation identity^a^		
Lesbian	23	19.2
Bisexual	73	60.8
Pansexual	18	15.0
Queer	17	14.2
Another sexual orientation identity	17	14.2
Gender identity^a^		
Man or transmasculine	5	4.2
Non-binary, agender, genderqueer, gender fluid, or questioning	17	14.2
Woman	110	91.7
Place of residence		
Rural	14	11.7
Suburban	52	43.3
Urban	54	45.0
Geographic region		
Midwest	14	11.7
Northeast	20	16.7
South	73	60.8
West	13	10.8
Educational attainment		
High school or less^b^	12	10.0
Some college or associate’s degree	59	49.2
Bachelor’s degree	41	34.2
Master’s degree or above	8	6.7
Employment status		
Student	27	22.5
Employed	70	58.3
Unemployed, not student	23	19.2
Health insurance status		
Uninsured	22	18.3
Insured	98	81.7
Usual place of care: yes	98	81.7
Ever experienced weight-based health care discrimination: yes	33	27.5
Ever received Pap test: yes	76	63.3
Received Pap test in last 3 years: yes	54	45.0

Percentages (%) are based on column totals and may not add to 100% due to rounding error.

^a^ Categories are not mutually exclusive.

^b^ High school diploma/general educational development or less than a high school degree.

We calculated univariate statistics for all study variables and cross-tabulated Pap test use (last 3 years and lifetime) and weight-based health care discrimination among Black SGM AFAB U.S. adults overall. We then used multivariable logistic modeling to estimate odds ratios (ORs) and 95% confidence intervals (CIs) for the associations between weight-based health care discrimination and Pap test use (last 3 years and lifetime), adjusting for demographic, socioeconomic, and health care factors. The proportion of missing data was ≤3%, with the exception of age (*n* = 17, 11%), which was imputed using a multiple imputation by chained equations algorithm that was run for five iterations.^54^ Participants missing age was included in the data set and analysis after imputation. Our analytic sample consisted of 120 respondents with no missing data for any study variable. All analyses were conducted using R version 4.3.2.^55^

## Results

The distribution of demographic, socioeconomic, and health care factors among Black SGM AFAB U.S. adults that could be recommended cervical cancer screening (*N* = 120) is presented in Table 1. The majority of respondents were between 21 and 29 years of age, lived in an urban or suburban area, resided in the South, had some college education or an associate’s degree, were employed, and had health insurance and a usual source of care. Approximately one quarter (27.5%) of respondents had ever experienced weight-based discrimination in a health care setting (Table 1). Moreover, we found that 63.3% and 45% of respondents had ever received a Pap test in their lifetime and in the last 3 years, respectively (Table 1). In particular, 48.5% of respondents who reported ever experiencing weight-based health care discrimination had received a Pap test in their lifetime relative to 69% of those who had not experienced this type of discrimination (data not shown). Similarly, Black SGM AFAB individuals exposed to weight-based discrimination in health care settings were significantly less likely to have obtained a Pap test in the last 3 years compared with their counterparts who had not been exposed to weight-based health care discrimination (30.3% vs. 50.6%; data not shown).

Table 2 shows that adjusting for demographic (i.e., age, place of residence, gender identity, sexual orientation identity, and geographic region) factors, Black SGM AFAB adults who had ever experienced weight-based discrimination in a health care setting had significantly lower adjusted odds of having received a Pap test in their lifetime (OR = 0.22; 95% CI: 0.06–0.69) and a Pap test in the last 3 years (OR = 0.33; 95% CI: 0.10–0.96) compared with those who had never experienced weight-based health care discrimination (Table 2, Model 2). Similarly, after adjusting for socioeconomic factors (i.e., educational attainment and employment status), we found that this effect became magnified; respondents who experienced weight-based health care discrimination had lower adjusted odds of having received a Pap test in their lifetime (OR = 0.11; 95% CI: 0.03–0.45) and a Pap test in the last 3 years (OR = 0.18; 95% CI: 0.05–0.63) compared with those who had never experienced weight-based health discrimination (Model 3). Finally, adjusting for health care factors (i.e., usual source of care, health insurance status), effects became more pronounced; respondents who had ever experienced weight-based health care discrimination had significantly lower adjusted odds of having received a Pap test in their lifetime (OR = 0.10; 95% CI: 0.02–0.40) and in the last 3 years (OR = 0.07; 95% CI: 0.01–0.31) compared with those who had never experienced such discrimination (Model 4).

**Table 2.** Odds of Pap Test Use Among Black Sexual Minoritized Cisgender Women, Transgender Men, and Non-Binary Assigned Female at Birth U.S. adults in Relation to Ever Experiencing Weight-Based Discrimination in a Health Care Setting (*N* = 120)

	Model 1	Model 2	Model 3	Model 4
	OR (95% CI)	OR (95% CI)	OR (95% CI)	OR (95% CI)
Ever received Pap test				
Ever experienced weight-based health care discrimination				
Yes	0.42 (0.18, 0.96)	0.22 (0.06, 0.69)	0.11 (0.03, 0.45)	0.10 (0.02, 0.40)
No (reference)	1.00	1.00	1.00	1.00
Received Pap test in last 3 years				
Ever experienced weight-based health care discrimination				
Yes	0.42 (0.17, 0.98)	0.33 (0.10, 0.96)	0.18 (0.05, 0.63)	0.07 (0.01, 0.31)
No (reference)	1.00	1.00	1.00	1.00

The 95% confidence intervals (CIs) for all ratios do not include 1. Model 1 is unadjusted. Model 2 is adjusted for age, place of residence, gender identity, sexual orientation identity, and geographic region only. Model 3 adds educational attainment and employment status to Model 2. Model 4 adds the usual source of care and health insurance status to Model 3.

OR, odds ratio.

## Discussion

We conducted the first study of which we are aware to examine the association between weight-based health care discrimination and cervical cancer screening among Black SGM AFAB adults. Specifically, we found that individuals who had experienced weight-based discrimination in health care settings had significantly lower odds of obtaining a Pap test in their lifetime and in the last 3 years relative to those who had not experienced weight-based health care discrimination, adjusting for demographic, socioeconomic, and health care factors. Our study extends the small literature on the negative association between weight-based health care discrimination and receipt of cervical cancer screening conducted among predominantly white women or non-SGM AFAB adults.^30,38,56,57^ Additionally, our findings contribute to the extremely limited literature on the impact of weight-based discrimination on Pap testing among Black SGM AFAB individuals, consisting of one qualitative study suggesting that the cervical cancer screening and other SRH experiences of Black and other transmasculine people of color are undermined by multiple forms of discrimination, including weight-based health care discrimination.^22^

The present study has important implications for addressing weight-based health care discrimination, specifically among Black SGM AFAB people and other multiply marginalized groups whose health care experiences are shaped by racism, sexism, heterosexism, and/or cisgenderism. First, health care institutions should cease promoting stigmatizing attitudes toward fat individuals,^34^ which providers are exposed to during professional training.^58,59^ Despite the proliferation of trainings and education to address multiple forms of oppression, these efforts have resulted in limited, and often temporary, changes for health care providers.^60–64^ One program for medical students addressing weight bias yielded no significant changes at the time of the study.^64^ These trainings alone fall short of changing how fat Black SGM patients are treated due to the pervasiveness of systemic racism, transphobia, and fatphobia in biomedicine and education.^33,34,65^

Second, health care providers, trainees, and institutions should move toward anti-oppressive, weight-inclusive approaches (e.g., Health At Every Size [HAES]); such frameworks reject the use of weight or BMI as measures of health.^66,67^ Important to note, however, is that fat activists and scholars have critiqued HAES and similar approaches for perpetuating oppressive systems (e.g., eugenics, white supremacy) that subjugate people living at the intersection of Blackness, gender, fatness, and disability.^33,36,68^ Addressing medical weight stigma thus requires a commitment from health care professionals to recognize and redress how weight discrimination intersects with multiple systems of oppression (e.g., ableism, racism, transphobia, heterosexism). This will involve reshaping harmful medical beliefs about fat Black SGM people,^33,34,36^ in addition to material commitments, such as investments in health care equipment (e.g., large tables and speculums), to promote the care of fat Black SGM people’s bodies *regardless* of perceived health status.^57^ Health care institutions must prioritize ensuring providers have the knowledge and resources to be competent in the care of fat bodies.

In particular, our study has important implications for facilitating access to Pap testing among Black SGM people facing weight-based discrimination in health care. At the interpersonal level, providers would benefit from prioritizing shared decision-making (SDM) to make care decisions that are aligned with patients’ specific preferences, needs, and values, such as when determining the appropriateness of discussing weight.^32,69^ SDM may improve the cervical cancer screening experiences and participation of Black SGM patients, particularly when clinicians use gender- and fat-affirming language and discuss comfort during Pap tests.^69,70^ Broadly, SRH can be improved among Black SGM people by hiring and supporting health care providers and other non-provider advocates supportive of Black SGM people experiencing weight-based discrimination and who can help patients effectively navigate health care institutions.^71,72^ This includes training and hiring more Black SGM providers underrepresented in health care,^73^ as studies indicate that patients receiving care from providers who share their marginalized identities report greater participation in their care and higher levels of patient satisfaction.^74,75^

Our findings should be interpreted in the context of several limitations. First, most participants had some college education, health insurance, and a usual source of care. As such, our results may not reflect Black SGM AFAB individuals with less access to these social, economic, and health care resources. Second, study participants eligible for a Pap test ranged from age 21 to 45, making findings less applicable to Black SGM AFAB adults over 45 eligible for cervical cancer screening. Third, our study’s small non-probability sample and cross-sectional design with self-reported Pap test use limit applicability to all Black SGM adults and introduce self-report bias into our findings. Additionally, having both the outcome variable “ever received Pap test” and the independent variable “ever experienced weight-based health care discrimination,” both of which were measured over a lifetime, makes it unclear whether discrimination happened before or after the screening, although the “received Pap test in last 3 years” variable may help reduce ambiguity. However, given the cross-sectional nature of our data, we are not able to establish temporality among study variables and do not make any causal claims about the associations between weight-based health care discrimination and Pap test use. As such, future research that uses longitudinal data where weight-based discrimination and cervical cancer screening are evaluated over time is warranted. Future research should also draw from large national probability sample surveys (e.g., National Survey of Family Growth) that oversample Black SGM AFAB people and include weight-based discrimination measures.

Fourth, our study did not investigate how weight-based health discrimination shapes Pap test use among Black SGM AFAB people in relation to other forms of discrimination that impact this multiply marginalized population, including racism, heterosexism, and cisgenderism, among others.^7–10,13–15,22,76,77^ Thus, future quantitative, qualitative, and mixed-methods research studies are needed to elucidate how multiple, intersecting forms of discrimination based on weight and other interconnected social categories (e.g., race/ethnicity, gender identity, sexual orientation, socioeconomic position, disability) shape Black SGM AFAB individuals’ cervical cancer screening experiences. Fifth, although the survey employed a validated tool (DMS) to measure discrimination in health care settings, the wording of the question makes it difficult to determine whether discrimination was experienced in interactions with providers, staff, or other patients. Thus, future research should develop and incorporate scales that allow researchers to parse out different sources of discrimination in health care settings. Last, the present study combined all subgroups of Black SGM people, a diverse and heterogeneous population in terms of race/ethnicity, sexual identity, and gender identity, among others. As such, future research should ensure the inclusion of large enough subgroups of Black SGM individuals to provide disaggregated estimates that reflect each subpopulation’s unique experiences.

### Health Equity Implications

Our study also has broader health equity implications for Black SGM AFAB people. First, organizational and institutional efforts are needed to improve access to cervical cancer screening and other SRH services for Black SGM AFAB.^7,9–16^ SDM is also influenced by the structure and operations of health care organizations.^69^ Organizations can improve their SDM capacity through shifts in health information technology, organizational structure and culture, physical environment, and incentives.^69,78^ They can integrate SDM into the electronic health record with culturally sensitive visit notes; improve the built environment, such as including adequate medical equipment for fat patients;^79^ and utilize financial and non-financial incentives to make SDM easier to incorporate,^69^ all of which can shift cultural and social norms about respecting the autonomy of fat Black SGM patients. Another way in which organizations can shift norms through SDM is to adopt options that improve autonomy, such as offering self-collection of vaginal samples for cervical cancer screening,^80^ addressing the specific barriers Black SGM AFAB people face due to heightened risk of violence and abuse.^33,34,81–83^ SDM is also improved when health care organizations embrace abolitionist practices.^69,71,84^ Abolition medicine advocates for universal health care access, reallocating resources from policing and other oppressive systems to services led by Black SGM community leaders, and creating health care spaces free from state or police presence.^84^

Additionally, it is crucial to acknowledge the health care system’s ongoing role in undermining the health of Black and SGM populations through medical experimentation, denial of care, and abuse.^33,34,82,83^ Therefore, facilitating Black SGM people’s access to care outside formal institutions is also necessary for health equity. For example, the Black Panther Party’s free clinics^85^ and the Young Lords’ community lead poisoning testing^86^ exemplify efforts to provide community care aligned with healing justice—a Black feminist framework emphasizing community-led care strategies beyond the medical industrial complex.^87^ Healing justice networks may improve care for fat Black SGM people by being grounded in community, an essential component of well-being for multiply marginalized people with historical trauma from the state and Western systems of care.^87^ Addressing weight-based health care discrimination and the barriers to care that it engenders among Black SGM AFAB adults will therefore require a holistic, intersectional, abolitionist, anti-oppressive approach that takes seriously their lived experiences in historical and social context and prioritizes healing, health, and well-being in this community by not only reforming existing health care organizations but by establishing and nurturing collective care networks outside of oppressive institutions^84,87^

## Abbreviations Used

AFAB: assigned female at birth
BMI: body mass index
CI: confidence interval
DMS: Discrimination in Medical Settings Scale
HAES: Health At Every Size framework
HC: health care
NSFG: National Survey of Family Growth
OR: odds ratio
SDM: shared decision-making
SE: socioeconomic
SGM: sexual and gender minoritized
SRH: sexual and reproductive health care
USPSTF: U.S. Preventive Services Task Force
